# Translational research of boron neutron capture therapy for spinal cord gliomas using rat model

**DOI:** 10.1038/s41598-024-58728-x

**Published:** 2024-04-09

**Authors:** Ryo Kayama, Kohei Tsujino, Shinji Kawabata, Yoshiki Fujikawa, Hideki Kashiwagi, Yusuke Fukuo, Ryo Hiramatsu, Takashi Takata, Hiroki Tanaka, Minoru Suzuki, Naonori Hu, Shin-Ichi Miyatake, Toshihiro Takami, Masahiko Wanibuchi

**Affiliations:** 1 Department of Neurosurgery, Osaka Medical and Pharmaceutical University, 2-7 Daigaku-Machi, Takatsuki City, Osaka, Japan; 2 Institute for Integrated Radiation and Nuclear Science, Kyoto University, 2 Asashiro-Nishi, Kumatori-Cho, Sennan-Gun, Osaka, Japan; 3 Kansai BNCT Medical Center, Osaka Medical and Pharmaceutical University, 2-7 Daigakumachi, Takatsuki City, Osaka, Japan

**Keywords:** Boron neutron capture therapy (BNCT), Boronophenylalanine, Spinal cord glioma, Particle radiation therapy, Rat model, Cancer, Drug discovery, Neurology, Oncology

## Abstract

Boron neutron capture therapy (BNCT) is a type of targeted particle radiation therapy with potential applications at the cellular level. Spinal cord gliomas (SCGs) present a substantial challenge owing to their poor prognosis and the lack of effective postoperative treatments. This study evaluated the efficacy of BNCT in a rat SCGs model employing the Basso, Beattie, and Bresnahan (BBB) scale to assess postoperative locomotor activity. We confirmed the presence of adequate in vitro boron concentrations in F98 rat glioma and 9L rat gliosarcoma cells exposed to boronophenylalanine (BPA) and in vivo tumor boron concentration 2.5 h after intravenous BPA administration. In vivo neutron irradiation significantly enhanced survival in the BNCT group when compared with that in the untreated group, with a minimal BBB scale reduction in all sham-operated groups. These findings highlight the potential of BNCT as a promising treatment option for SCGs.

## Introduction

Boron neutron capture therapy (BNCT) is a treatment strategy for unresectable refractory tumors. Its key advantage lies in its ability as particle irradiation therapy, facilitating the selective destruction of tumor cells. During clinical BNCT, patients are intravenously administered a boron compound that accumulates within tumor cells, which is followed by neutron irradiation. The exposure of tumor cells enriched with boron-10 to low-energy thermal neutrons elicits the production of high linear energy transfer (LET) particles. The particles path length is approximately 10 μm, which is approximately equal to the cell diameter. Consequently, the cell-killing effect of high-LET particles is limited to tumor cells containing boron-10^1–3^. In order to take advantage of this property, BNCT has been applied as a treatment method for various intractable tumors^4^ and recurrent tumors^5,6^. Given its promising outcomes in nuclear reactors, BNCT could be developed as a treatment for invasive cancers such as high-grade meningiomas^7,8^ and high-grade gliomas^1,9,10^. The successful development of an accelerator neutron source has demonstrated the effectiveness of BNCT in high-grade gliomas^5^. Currently, BNCT using boronophenylalanine (BPA) for head and neck cancer is covered by insurance in Japan^11^. Accelerator-based BNCT medical devices can be easily installed in hospitals, highlighting their potential for widespread use in clinical practice^5,11^.

Primary spinal cord tumors are rare, representing 2–4% of central nervous system tumors, with intramedullary spinal cord tumors (IMSCTs) constituting 10% of this category. Spinal cord gliomas (SCGs) account for 10% of IMSCTs^12–16^. Among intramedullary spinal tumors (IMSTs), ependymomas and astrocytomas are the most prevalent glial tumors derived from glial cells^17^. Although most ependymomas are benign and low-grade (World Health Organization grade I or II), a subset is classified as WHO grade III–IV. Approximately 25% of astrocytomas are also categorized as high-grade (WHO III–IV)^14^. Similar to intracranial gliomas, SCGs are associated with poor prognosis. One study targeting primary anaplastic astrocytoma and glioblastoma reported an extremely poor prognosis with a 5-year survival rate of 18.7% and a 10-year survival rate of 13.8%^18^.

Owing to their invasive nature, the treatment of SCGs requires targeting not only the tumor mass but also the surrounding invasive regions. In accordance with the management of intracranial gliomas, interventions for IMSCTs involve surgical resection, radiation therapy (X-ray or γ-ray), and chemotherapy. However, given their rarity, accumulated evidence regarding SCGs remains elusive, and the optimal management strategy remains controversial despite the poor prognosis^19^. While most patients with SCG undergo surgical resection as their initial course of treatment, achieving gross total removal of high-grade SCGs is often challenging and leads to inevitable surgical complications. Additionally, previous reports have indicated that the extent of removal does not substantially impact the prognosis^18^. Furthermore, a report that evaluated postoperative neurological function in anaplastic astrocytoma and glioblastoma using the modified McCormick score found that postoperative neurological function deteriorated in many cases^20^. Many patients with SCG tend to receive radiotherapy (X-ray or γ-ray) as a postoperative adjuvant therapy, and irradiation planning with fewer adverse events is feasible^21^. However, there is no consensus on the effectiveness of radiotherapy (X-ray or γ-ray), as there are opposing opinions^18,22–24^.Moreover, in cases of recurrence after radiation therapy, options are limited by the tolerated radiation dose, with no effective treatment currently available^25,26^. Although chemotherapy such as temozolomide and bevacizumab has been proposed to be effective in treating spinal cord high-grade glioma, chemotherapy has failed to elicit considerable survival benefits^27,28^. Consequently, effective treatments for malignant SCGs are currently lacking, necessitating the development of novel treatment modalities.

In the historical context of BNCT clinical trials, the initial phase involved neutron irradiation using BPA or borocaptate sodium to the spinal cord to assess the tolerance of normal tissues to treatment^29–34^. Both the brain and the spinal cord, parts of the central nervous system, exhibit similar post-irradiation late effects, characterized by selective necrosis of the white matter. However, in terms of late effects, the endpoint of irradiation of the spinal cord is more precisely defined than that of the brain, given the occurrence of radiation myelopathy^32^. Consequently, neutron irradiation of the spinal cord of a rat has frequently been employed to evaluate the impact of BNCT radiation on the central nervous system, and these findings have been subsequently applied to BNCT for intracranial gliomas.

Notably, the primary objective of the majority of previous reports was not the treatment of spinal cord tumors but rather the assessment of BNCT safety for central nervous system tissues and its potential clinical application in brain tumors. In contrast, the current study focused on the treatment of SCGs, marking a departure from the goals of the previous studies. Furthermore, in earlier BNCT experiments, there was no consensus regarding the radiation myelopathy threshold. However, nowadays, a more defined consensus on the appropriate dose to prevent radiation myelopathy has been established. Conventional stereotactic radiotherapy to the spinal cord is commonly administered at a total dose of 50 Gy in 25 fractions, with an incidence of radiation myelopathy of less than 0.1%^21^. Some recommendations have established a safe maximum dose limit for a single irradiation. Armed with these foundational data, simulating BNCT for human intramedullary spinal cord tumors is markedly more realistic than in the past. Moreover, previous studies on the tolerable dose to the rat spinal cord have provided valuable insights for designing irradiation experiments in rat disease models^32,35^.

Based on these research backgrounds, we considered it was plausible to estimate the applicability of BNCT for SCGs Most studies involving BNCT for central nervous system tumors have focused on intracranial malignant gliomas. Our previous reports have demonstrated the effectiveness of reactor- and accelerator-based BNCT for intracranial malignant gliomas^5,9^. The application of BNCT to SCGs represents a novel approach that could address the challenges posed by aggressive and hard-to-treat tumors. In the current study, we aimed to assess the efficacy of BNCT against SCGs using a rat SCG model. To the best of our knowledge, this is the first report to evaluate the efficacy of in vivo BNCT in a SCG model.

## Results

### Cellular uptake and retention of boron in F98 glioma and 9L gliosarcoma cells following BPA exposure

**Figure 1:**
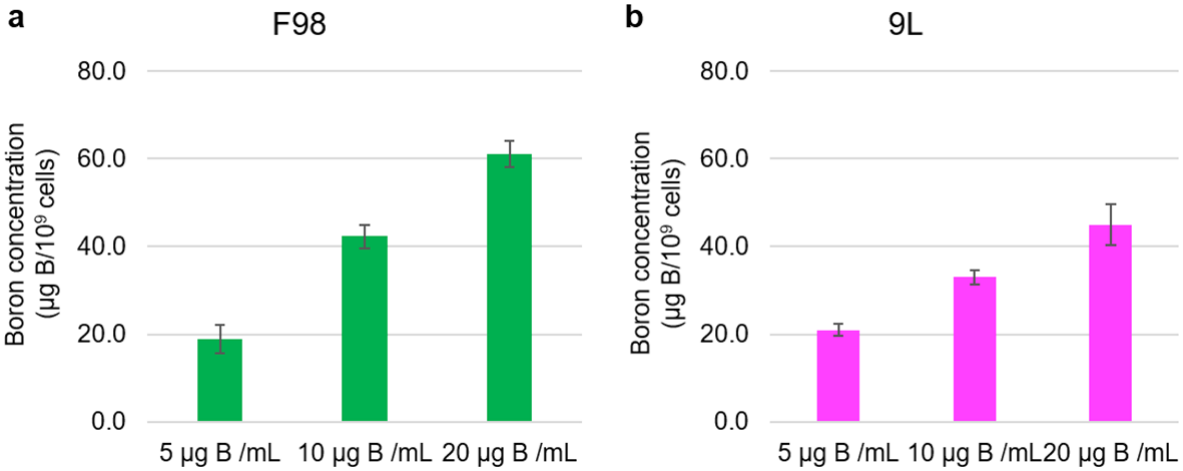
In vitro cellular uptake of boron in F98 rat glioma and 9L rat gliosarcoma cell. (**a**,**b)** Cellular boron concentrations of each cell line exposed to BPA 5,10, or 20 μg B/mL for 2.5 h are shown (**a**: F98, **b**: 9L). The bar in each result indicates the standard deviation (SD, n = 3). The boron concentration is defined as μg B/10^9^ cells. Statistical analysis was performed using Student’s *t*-test.

Boron concentrations in F98 and 9L cells following exposure to various BPA concentrations are shown in Fig. 1a-b.

After incubation in 5 µg 10B/mL BPA for 2.5 h, boron concentrations of 19.0 ± 3.2 and 21.0 ± 1.3 µg 10B/10^9^ cells were detected in F98 and 9L cells, respectively (*P* = 0.47). Incubation in 10 µg 10B/mL BPA for 2.5 h resulted in boron concentrations of 42.3 ± 2.6 and 33.1 ± 1.6 µg 10B/10^9^ cells (*P* = 0.01), while incubation with 20 µg 10B/mL BPA for 2.5 h yielded concentrations of 61.0 ± 2.9 and 45.0 ± 4.5 µg 10B/10^9^ cells (*P* = 0.01) in F98 and 9L cells, respectively. In both cell types, the cellular uptake of boron increased with increasing BPA concentrations at 2.5 h of exposure (F98 cells: 5 μg 10B/mL vs. 10 μg 10B/mL, *P* < 0.001; 5 μg 10B/mL vs. 20 μg 10B/mL, *P* < 0.0001; and 10 μg 10B/mL vs. 20 μg 10B/mL, *P* < 0.001. 9L cells: 5 μg 10B/mL vs. 10 μg 10B/mL, *P* < 0.01, 5 μg 10B/mL vs. 20 μg 10B/mL, *P* < 0.001 and 10 μg 10B/mL vs. 20 μg 10B/mL, *P* < 0.001).

### Evaluation of survival rate and BBB scale in F98 and 9L rat SCG models

**Figure 2:**
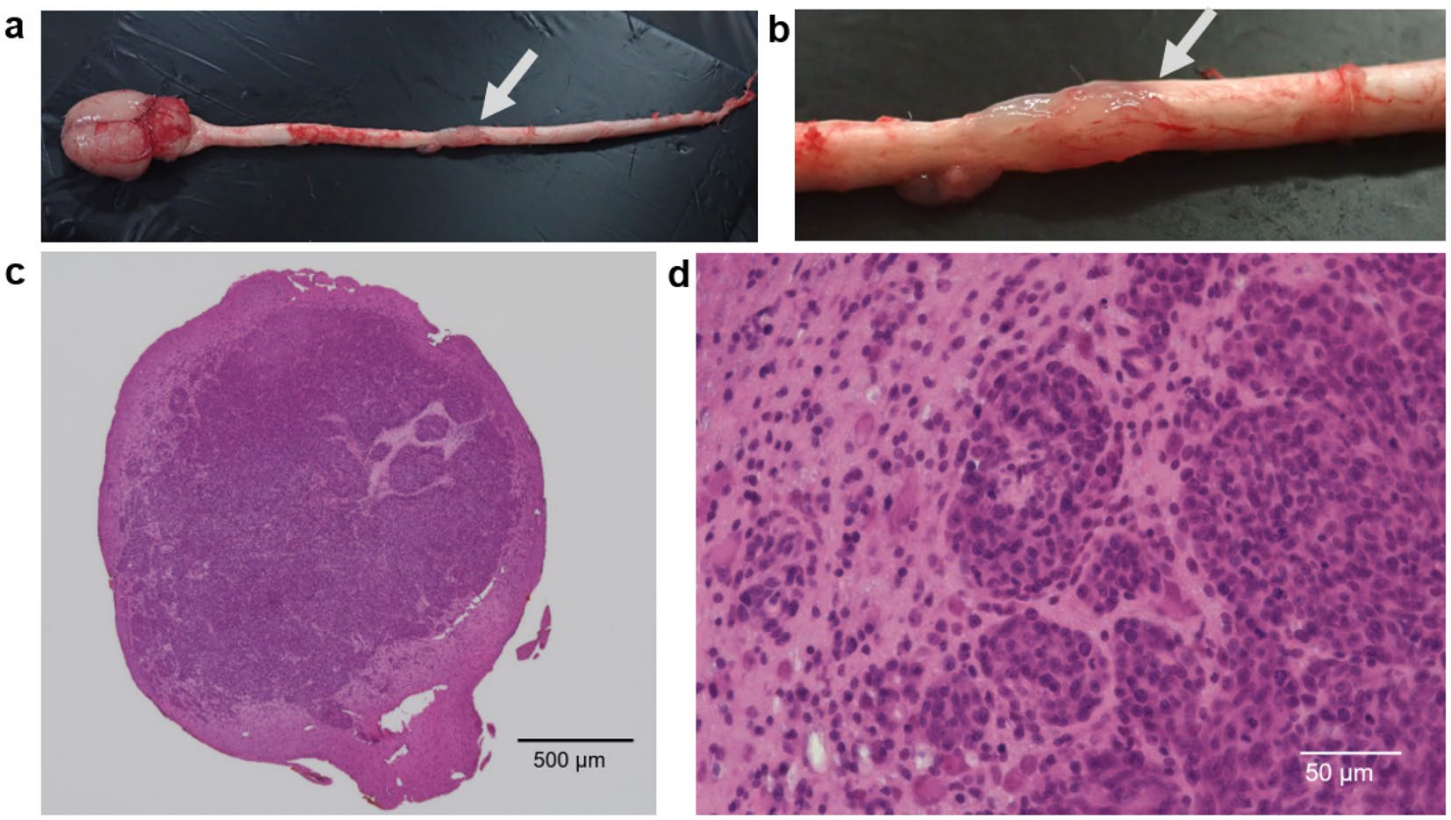
F98 rat spinal cord glioma model. (**a**) Central nervous system organ specimens including brain and spinal cord of F98 rat spinal cord tumor model. The arrow in the figure indicates the F98 spinal cord tumor mass at Th 9/10 levels. (**b**) This figure shows the F98 spinal cord tumor mass enlarged figure (**a**). The arrow in the figure indicates the F98 spinal cord tumor mass at Th 9/10 levels. (**c**) This figure shows the representative aspects of F98 rat spinal cord tumor sections stained hematoxylin Eosin (H-E)(× 4). This sections removed from rats sacrificed after implantation of F98 rat glioma cells, in which the BBB scale was 5 or less. Scale bar indicates 500 μm. (**d**) This figure shows the area of rat spinal cord infiltrated by F98 rat glioma cells (×40). Scale bar indicates 50 μm.

**Figure 3:**
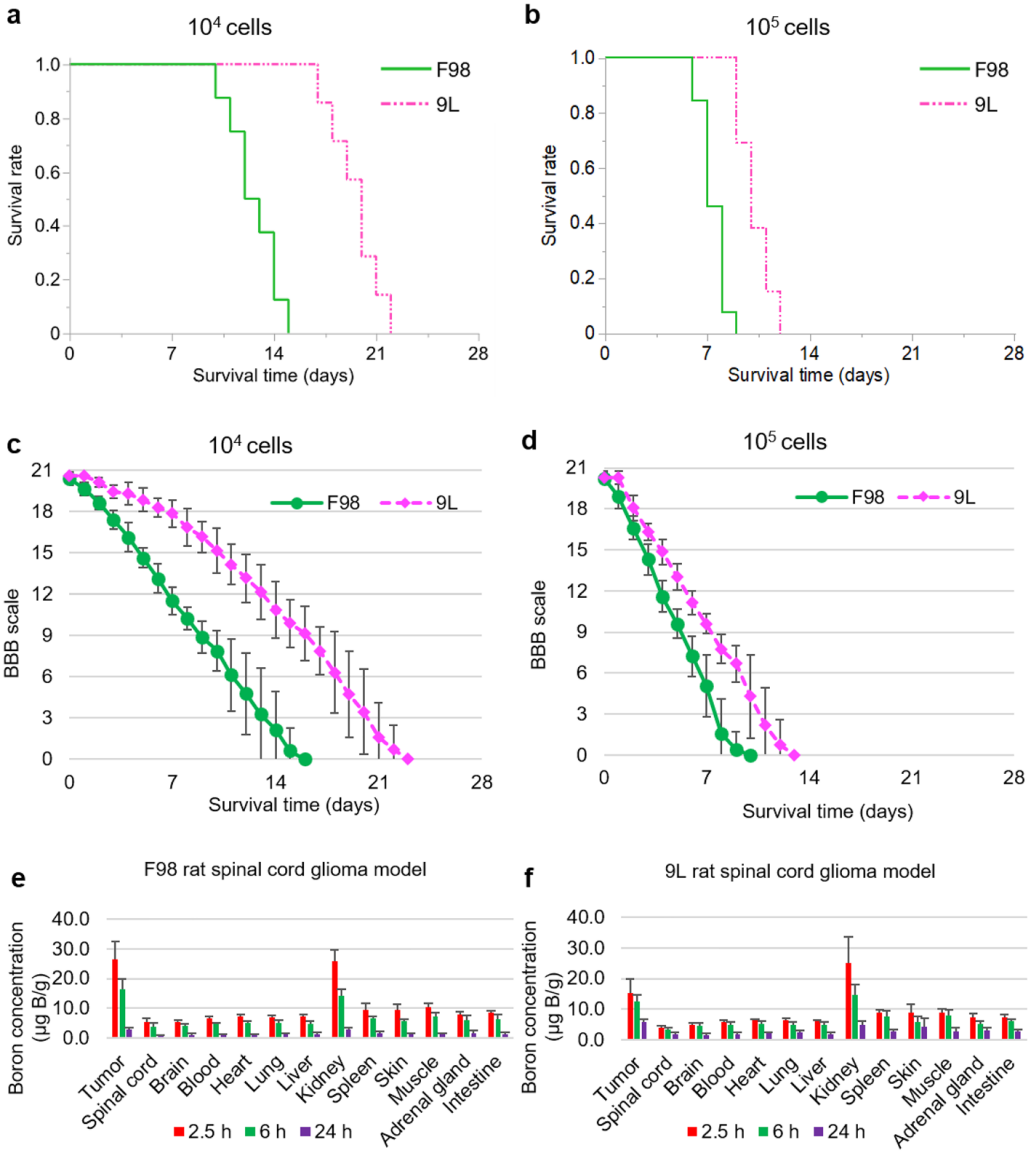
Kaplan–Meier curves, the BBB scale and the in vivo biodistribution of boron of F98 and 9L SCG models. (**a**,**b**) These Kaplan–Meier curves show the survival time of F98 or 9L rat SCG models after implantation of each cell line (a; 10^4^ cells, b; 10^5^ cells). Survival times (days) are plotted. Four groups were prepared; F98 10^4^ cells (n = 8), F98 10^5^ cells (n = 13), 9L 10^4^ cells (n = 7), and 9L 10^5^ cells (n = 13). Statistical analysis was performed using the log rank test. (**c**,**d**) These figures show the BBB scale in the case of implantation of F98 or 9L and 10^4^ cells or 10^5^ cells. The error bar indicates the standard deviation (SD). Statistical analysis was performed using the multivariate analysis of variance (MANOVA). **e–f,** These figures show the time course of boron concentration in each organ after intravenous administration of BPA (250 mg /kg b.w.) to the F98 or 9L rat SCG models (e; F98, f; 9L). All the bars indicate standard deviation (SD; F98 2.5 h: n = 8, 6 h: n = 4, 24 h: n = 4, 9L 2.5 h: n = 6, 6 h: n = 4, 24 h: n = 4).

F98 and 9L SCG models were successfully generated (Fig. 2a-d). Figure 3a-d and Table 1 present the Kaplan–Meier curves and the Basso, Beattie, and Bresnahan (BBB) scale evaluations following implantation of each cell line (10^4^ or 10^5^ cells). The median survival time for each condition was as follows: F98 10^4^ cells, 12.5 days (95% confidence interval [CI] 10–14 days); F98 10^5^ cells, 7.0 days (95% CI 6–9 days); 9L 10^4^ cells, 20.0 days (95% CI 17 –22 days); and 9L 10^5^ cells, 10.0 days (95% CI 9–12 days) (F98 10^4^ cells *vs.* F98 10^5^ cells, *P* < 0.0001; 9L 10^4^ cells *vs.* 9L 10^5^ cells, *P* < 0.001; F98 10^4^ cells *vs.* 9L 10^4^ cells, *P* = 0.0001; F98 10^5^ cells *vs.* 9L 10^5^cells, *P* < 0.0001 ). The BBB scale scores decreased gradually in each group (F98 10^4^ cells *vs*. F98 10^5^ cells, *P* < 0.0001; 9L 10^4^ cells *vs*. 9L 10^5^ cells, *P* < 0.0001; F98 10^4^ cells *vs*. 9L 10^4^ cells, *P* < 0.0001; F98 10^5^ cells *vs*. 9L 10^5^ cells, *P* < 0.0001 ).

**Table 1:** Survival times of F98 or 9L rat SCG models after implantation of each tumor cell.

Group	n^a^	Survival times (days)		
		Mean ± SD	Median	95% CI^b^
F98 10^4^ cells	8	12.6 ± 1.6	12.5	10–14
F98 10^5^ cells	13	7.4 ± 0.8	7	6–9
9L 10^4^ cells	7	19.6 ± 1.6	20	17–22
9L 10^5^ cells	13	10.2 ± 1.0	10	9–12

^a^n indicates the number of Fischer rats per group.

^b^CI is confidential interval.

For the therapeutic neutron irradiation experiment, glioma rat spinal cord tumor models implanted with 10^4^ cells were used owing to the excessively rapid symptom progression in the SCG models implanted with 10^5^ cells.

### In vivo biodistribution of boron in F98 and 9L rat SCG models

Table 2 presents the boron concentrations in the tumor, spinal cord, brain, and blood at 2.5, 6, and 24 h after the intravenous (i.v.) BPA administration in the two rat spinal cord glioma models. In F98 rat SCG models, boron concentrations in the tumor, spinal cord, brain, and blood were as follows: at 2.5 h after i.v. BPA, 26.6 ± 5.8, 5.3 ± 1.3, 5.5 ± 0.5, and 6.7 ± 0.7 μg 10B/g; at 6 h after i.v. BPA, 16.3 ± 3.6, 4.0 ± 1.1, 4.2 ± 0.6, and 4.7 ± 0.6 μg 10B/g; at 24 h after i.v. BPA, 3.0 ± 0.4, 0.8 ± 0.2, 1.1 ± 0.5, and 1.1 ± 0.2 μg 10B/g, respectively. Additionally, in 9L rat SCG models, boron concentrations in the tumor, spinal cord, brain, and blood were as follows: at 2.5 h after i.v. BPA, 15.3 ± 4.5, 4.2 ± 0.6, 5.0 ± 0.7, and 5.9 ± 0.5 μg 10B/g; at 6 h after i.v. BPA, 12.6 ± 2.3, 3.3 ± 0.7, 4.7 ± 1.0, and 5.0 ± 0.9 μg 10B/g; at 24 h after i.v. BPA, 6.0 ± 0.8, 2.0 ± 0.7, 1.8 ± 0.6, and 1.9 ± 0.5 μg 10B/g, respectively.

**Table 2:** The summary of the boron concentrations in each tissue of the F98 and 9L rat SCG models.

Cell line	Boron compound (dose)	Time^a^ (h)	n^b^	Boron concentrations ± SD (μg B/g)				Ratios		
				Tumor	Spinal cord	Brain	Blood	T/SC^c^	T/Bl^d^	Bl/SC^e^
F98 rat glioma	BPA (250 mg/kg)	2.5	8	26.6 ± 5.8	5.3 ± 1.3	5.5 ± 0.5	6.7 ± 0.7	5.0	4.0	1.3
		6	4	16.3 ± 3.6	4.0 ± 1.1	4.2 ± 0.6	4.7 ± 0.6	4.1	3.5	1.2
		24	4	3.0 ± 0.4	0.8 ± 0.2	1.1 ± 0.5	1.1 ± 0.2	3.6	2.8	1.3
9L rat gliosarcoma		2.5	6	15.3 ± 4.5	4.2 ± 0.6	5.0 ± 0.7	5.9 ± 0.5	3.7	2.6	1.4
		6	5	12.6 ± 2.3	3.3 ± 0.7	4.7 ± 1.0	5.0 ± 0.9	3.8	2.5	1.5
		24	4	6.0 ± 0.8	2.0 ± 0.7	1.8 ± 0.6	1.9 ± 0.5	3.0	3.2	1.0

^a^Time indicates the time after the termination intravenous administration.

^b^n indicates the number of the Fischer rats.

^c^T/SC: Tumor to Spinal cord (SC) ratio.

^d^T/Bl: Tumor to Blood ratio.

^e^Bl/SC: Blood to Spinal Cord (SC) ratio.

In both rat SCG models, boron concentrations in all organs gradually decreased from 2.5 to 24 h. Figure 3e and 3f illustrate the boron concentrations in each organ. Based on these results (absolute boron uptake, T/Bl and T/SC ratios), neutron irradiation studies were performed at 2.5 h after i.v. BPA.

### Therapeutic efficacy of BNCT in F98 rat SCG models

**Figure 4:**
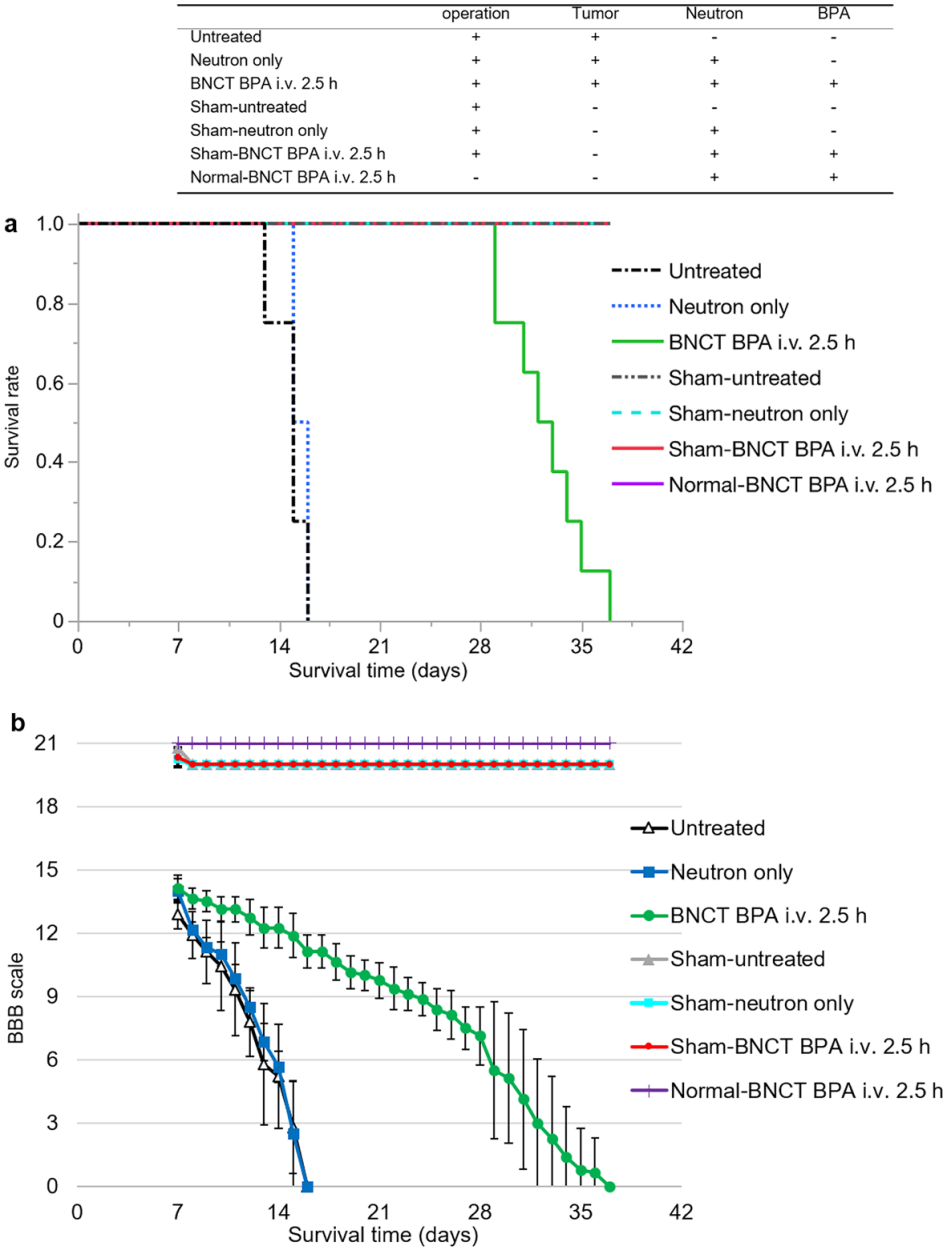
Kaplan–Meier curves and the BBB scale in the in vivo neutron irradiation of F98 rat SCG models, sham-operated models and normal rats. (**a**) This figure shows the results of the Kaplan–Meier curves in the in vivo neutron irradiation for F98 rat SCG models and the sham-operated models. Survival times (days) are plotted. Statistical analysis was performed using the log rank test (Untreated: n = 8, Neutron only: n = 6, BNCT BPA i.v. 2.5 h: n = 8). (**b**) This figure shows the results of the BBB scale in the in vivo neutron irradiation for F98 rat SCG models, the sham-operated models, and normal rats. BBB scales are plotted. In BBB scale, the evaluation was started at the day of neutron irradiation (7 days after implantation of F98 glioma cells.). The error bar indicates the standard deviation (SD). Statistical analysis was performed using the multivariate analysis of variance (MANOVA).

Figure 4a presents the Kaplan–Meier survival curves, and Fig. 4b illustrates the BBB scale results for each group. In the in vivo neutron irradiation experiment performed in the F98 rat SCG model (Study 1), we noted significant differences (*P* < 0.0001) in terms of survival between the untreated and BNCT BPA 2.5 h i.v. groups using the log-rank test. Additionally, multivariate analysis identified significant differences (*P* < 0.0001) in terms of BBB scale reduction between the untreated and BNCT BPA 2.5 h i.v. groups. The median survival time (MST) and percentage increase in lifespan (%ILS) are shown in Table 3. In the in vivo neutron irradiation experiment performed in the sham-operated group (Study 2), all rats survived until 37 days after the sham operation, with no significant decrease in their BBB scale. Furthermore, after in vivo neutron irradiation of normal rats (Study 3), the BBB scale score for all rats remained unchanged.

**Table 3:** Survival times of the F98 rat SCG models after neutron irradiation.

Group	n^a^	Survival times (days)			%ILS^c^	*p*-value^d^
		Mean ± SD	Median	95% CI^b^		
Untreated	8	14.8 ± 1.1	15	13–16	–	–
Neutron only	6	15.5 ± 0.5	15.5	15–16	3.3	0.224
BNCT BPA i.v. 2.5 h	8	32.5 ± 2.6	32.5	29–35	116.7	< 0.0001

^a^n indicates the number of Fischer rats per group.

^b^CI is confidence interval.

^c^Percent increase in life span (%ILS) was defined relative to the mean survival time (MST) of the untreated group.

^d^*p*-values were calculated using the log-rank test compared to untreated based on the results obtained from the Kaplan–Meier curves in vivo neutron irradiation.

### Dosimetric evaluation of in vivo neutron irradiation during BNCT for spinal cord tumors

The absorbed and photon-equivalent doses for in vivo neutron irradiation are listed in Table 4. Compound biological effectiveness (CBE) of 3.8 and 1.35 was applied for spinal cord tumors and the normal spinal cord, respectively^9,36^. Relative biological effectiveness (RBE_N_ and RBE_H_) values were set at 3.0, as determined using a previous report^37^. Following BNCT BPA i.v., the photon-equivalent dose for spinal cord tumor was 19.5 Gy-Eq at 2.5 h, while the equivalent dose for the normal spinal cord was 3.5 Gy-Eq.

**Table 4:** The estimated absorbed dose and the photon-equivalent dose in the in vivo neutron irradiation.

Group	Absorbed dose^a^ (Gy)		Photon-equivalent dose^b^ (Gy-Eq)	
	Spinal cord	Tumor	Spinal cord	Tumor
Untreated	–	–	–	–
Neutron only	1.2	1.2	2.3	2.3
BNCT BPA i.v. 2.5 h	2.2	5.8	3.5	19.5
Sham-untreated	-	-	-	-
Sham-neutron only	1.4	-	2.4	-
Sham-BNCT BPA i.v. 2.5 h	2.3	-	3.6	-

^a^The absorbed dose is attributed to the ^10^B(n,α)^7^Li, ^14^N(n,p)^14^C, and ^1^H(n,n)^1^H reactions produced by thermal, epithermal, and fast neutron fluxes and gamma rays in the irradiation neutrons. It is calculated using the following equation: absorbed dose (Gy) = D_B_ + D_N_ + D_H_ + D_γ_.

^b^The estimated photon-equivalent dose was calculated using the following equation: D_B_ × compound biological effectiveness + D_N_ × relative biological effect of nitrogen (RBE_N_) + D_H_ × relative biological effect of hydrogen (RBE_H_) + D_γ_. RBE_N_ and RBE_H_ are 3.0. In the case of BPA, the CBE factor for the normal spinal cord was adopted to 1.35, and for the tumor was 3.8.

## Discussion

In this study, we conducted in vitro experiments on F98 and 9L and in vivo experiments on the F98 rat SCG models, with the aim of applying BNCT to spinal cord malignant glioma, which has a poor prognosis.

According to in vitro cellular uptake assessment, sufficient boron concentrations were detected in BPA-exposed F98 and 9L cells (Fig. 1a, 1b), confirming a concentration-dependent increase in boron concentration upon BPA exposure. The in vivo biodistribution experiments revealed that the boron concentrations in the tumor 2.5 h after intravenous BPA administration were sufficient and gradually decreased over time (Table 2). BPA is taken up by tumor cells via L-type amino acid transporter 1 (LAT-1)^38^, which is highly expressed in tumor cells. In F98 glioma rat brain tumor models, a tumor-to-normal brain (T/N) ratio of ~ 3–4 has been reported^39–42^. Similarly, the present study focused on spinal cord tumor models, and the T/N (spinal cord tumor-to-normal spinal cord) ratio was also ~ 3–5, with nearly equivalent boron concentrations detected in the normal brain and normal spinal cord (Table 2). This finding suggests that BPA reaches tumor cells in the central nervous system, such as the cerebrospinal cord, via LAT-1, similar to the brain tumor model.

The in vivo neutron irradiation experiment in the sham surgery groups initially demonstrated minimal or no decrease in the BBB scale due to sham surgery. In normal rats, BNCT with BPA also exhibited no obvious decrease in the BBB scale score during the observation period, confirming the safety of BNCT. An in vivo neutron irradiation experiment using the F98 rat SCG model validated the efficacy of BNCT. The BNCT BPA i.v. 2.5 h group exhibited significantly prolonged survival when compared with the untreated and neutron-only groups. Furthermore, the BBB scale reduction in the BNCT BPA i.v. 2.5 h group was markedly lower than that in the untreated and neutron-only groups. The estimated photon-equivalent dose to the spinal cord tumor was 19.5 Gy-Eq, whereas the normal spinal cord was 3.5 Gy-Eq, indicating that the tumor selectively of BPA minimized damage to normal tissue and concentrated the neutron irradiation dose to the tumor. With a single dose of 12.4–14.0 Gy, the risk of radiation myelopathy is estimated to be 1–5%^25^. Thus, if such a dose gradient between tumor and normal spinal cord can be achieved in patients with SCG, it is anticipated that BNCT will be a highly effective treatment due to its safety as well as its therapeutic effects. Furthermore, according to Morris et al., although the dose and route of BPA administration are different, the ED50 for myelopathy after neutron irradiation was 14.7 ± 0.4 Gy after i.p. administration of BPA and 12.9 ± 0.3 Gy in rats irradiated after a 6-h i.v. infusion of BPA^43^. It is assumed that myelopathy hardly occurred in the BNCT BPA i.v. 2.5 h group. In the neutron-only group, the photon-equivalent dose to the tumor was 2.3 Gy-Eq, and the survival time of the BNCT BPA i.v. 2.5 h group was significantly extended when compared with that of the neutron-only group (*P* < 0.001), underscoring the high effectiveness of BNCT with BPA over neutron irradiation alone. The key attributes of BNCT, i.e., reduced damage to normal tissue and tumor-selective treatment, are advantageous in SCGs, as well as in intracranial malignant gliomas. BNCT shows a great deal of promise in treating SCGs, particularly those with limited options in terms of tolerable doses to the spinal cord post-radiotherapy.

Considering the clinical application of BNCT for SCGs, a distinctive advantage emerges when compared with the application of BNCT for brain tumors. Notably, depth variation, which is often encountered in the context of BNCT for brain tumors, is less relevant for spinal cord tumors. The variable depths at which brain tumors can occur pose a challenge, as obtaining a sufficient dose for therapeutic efficacy may be hindered, especially considering tumors located deep within the brain. Conversely, SCGs are more promising candidates than brain tumors for BNCT effectiveness, given their relatively uniform distribution and proximity to the body surface. Furthermore, BNCT offers the unique advantage of minimizing the risk of normal tissue damage owing to the heightened tumor selectivity for BPA, a characteristic intrinsic to BNCT. With careful dose planning, safe re-irradiation may be possible even in cases where radiation has been administered previously, thereby expanding the available therapeutic options.

However, the potential effect of BNCT with BPA on other organs within the irradiation field remains debatable. Herein, irradiation targeted a tumor at the thoracic spine level, necessitating the consideration of BNCT-mediated effects on the surrounding organs during the practical application of BNCT in clinical settings. A report on a phase II trial of BNCT for head and neck cancer concluded that grade 3 or higher adverse event was rare and that it was a safe treatment^11^. However, if we assume that both sides of the spine are shielded when irradiating the cervical spinal cord or thoracic spine with neutron beams, mucositis in the esophagus located on the ventral side of the spine may become a troublesome adverse event. In a case report of a lung tumor patient who underwent BNCT, it was reported that while the treatment showed a certain level of therapeutic effect, no adverse events exceeding grade 2 occurred^44^. In this report, it is speculated that radiation pneumonitis occurred in areas exposed to more than 4 Gy-Eq, but with sufficient shielding, it is unlikely that such an amount of exposure would occur. Nevertheless, caution should be exercised when applying BNCT to recurrent cases post-radiotherapy, given the naturally elevated risk of radiation exposure compared with initial episode cases.

In the current study, BNCT did not elicit a complete cure in any treated rat during the therapeutic experiment. Nevertheless, BNCT can be safely performed in normal rats. Future research should undertake additional experiments to demonstrate the efficacy of BNCT in SCGs. This may involve increasing the photon-equivalent dose to the tumor through dose planning or by extending the neutron irradiation time.

## Conclusions

Based on the findings of this translational study using a rat SCG model, BNCT holds promise as a viable therapeutic option for SCGs. Moreover, these findings underscore the need for further research to explore and establish clinical applicability. In conclusion, radiotherapy, although effective, addresses the challenges of sparing normal tissue and achieving curative doses. BNCT, with its high LET and cell-selective attributes, attracts attention as an optimal treatment modality, especially for cancers affecting radiosensitive organs such as the spinal cord. Ongoing progress in BNCT, particularly with the development of accelerator neutron sources, has resulted in substantial strides toward addressing these challenges in cancer treatment.

## Methods

### Boron compound

Herein, BPA (L-isomer) was purchased from Interpharma Praha (Prague, Czech Republic), converted into fructose complex^45^, and used as 1000 µg 10B/mL solution in the experiments. This boron compound was prepared as a boron-10-enriched compound.

### Cell culture

In vitro experiments were performed using two tumor cell lines: F98 rat glioma cells and 9L rat gliosarcoma cells^46^. F98 rat glioma cells were kindly provided by Dr. Rolf Barth (Ohio State University, Columbus, OH, USA), and 9L rat gliosarcoma cells were purchased from the American Type Culture Collection (Manassas, VA, USA)^41,42^. All cell lines were cultured in Dulbecco’s Modified Eagle’s Medium (DMEM), supplemented with 10% fetal bovine serum and penicillin, streptomycin, and amphotericin B at 37℃ under 5% CO_2_. All materials used in the culture medium were purchased from Gibco (Grand Island, NY)^39–42^. Of the two cell lines, F98 rat glioma cells were used in our laboratory for basic research on BNCT for intracranial malignant glioma^39–42^. F98 rat glioma cells can simulate the behavior of human malignant gliomas during intracerebral implantation, including their highly infiltrative growth pattern and low immunogenicity^46,47^. Therefore, we came up with the idea of creating a rat spinal cord tumor model in which F98 rat glioma cells are implanted into the spinal cord, which is also the central nervous system, and using this model to test the efficacy of BNCT. In the current study, F98 glioma or 9L gliosarcoma—bearing rat spinal cord tumor models were used to perform the in vivo experiment, named as “F98 rat SCG models” or “9L rat SCG models”.

### In vitro cellular uptake of boron in F98 rat glioma and 9L rat gliosarcoma cells

Cellular boron uptake by F98 glioma and 9L gliosarcoma cells exposed to BPA was examined in vitro. Initially, cells at a density of 5.0 × 10^5^ were seeded in 100-mm dishes (Becton, Dickinson, and Company, Franklin Lakes, NJ, USA) and incubated at 37℃ under a 5% CO_2_ atmosphere using the above-described culture medium^39,41,42^. After three days of incubation, the culture medium was replaced with a culture medium containing 5, 10, or 20 µg B/mL BPA and incubated for 2.5 h under the same conditions. Exposure was terminated by discarding the culture medium in half of the dishes, and the cells were washed with phosphate-buffered saline (PBS) at 4℃. The remaining dishes were replaced with a boron-free culture medium and incubated for 3.5 h. These cells were collected by centrifugation (at 200 × *g* for 5 min), followed by thorough agitation with 4 mL of PBS and subsequent enumeration. The cells were then lysed overnight in 1N nitric acid solution^39,41,42^. The amount of boron in the cell lysate was measured using inductively coupled plasma atomic emission spectroscopy (ICP-AES; iCAP6300 emission spectrometer, Hitachi, Tokyo, Japan). The boron concentrations in cells were defined as μg boron (10B)/10^9^ cells.

### F98 and 9L rat spinal cord glioma (SCG) models and rat medium injection models

This study was carried out in compliance with the ARRIVE guidelines. All animal experiments complied with “the Guide for the Care and Use of Laboratory Animals”, and were approved by both facilities: the Animal Use Review Board and Ethical Committee of Osaka Medical and Pharmaceutical University (No. 21124-A) and the Institute for Integrated Radiation and Nuclear Science, Kyoto University (KURNS; Kumatori, Osaka, Japan) (No.2022–23).

Herein, we employed 7-week-old male Fischer rats (Japan SLC, Shizuoka, Japan) weighing ~ 150–200 g. Rats were anesthetized by administering a mixture of anesthetics intraperitoneally: 0.4 mg/kg medetomidine (ZENOAQ, Fukushima, Japan), 2.0 mg/kg midazolam (SANDOZ, Yamagata, Japan), and 5.0 mg/kg butorphanol (Meiji Seika, Tokyo, Japan)^39–42^.

The rats were placed in a prone position on the operating table. The spinous process of thoracic (Th)12 was confirmed on the body surface. Thus, a ~ 4-cm skin incision was made in a cephalic-caudal direction, with the Th12 spinous process as the caudal side. The operative field was secured with an open wound, and the spinal muscle group was dissected from the spinous process to expose the vertebral arch^48,49^. For tumor implantation into the intramedullary spinal cord or sham operation at the Th9/10 level, the Th8 and Th10 spinous processes were fixed using an SR-5 M-HT + STS-A stereotactic frame (NARISHIGE, Tokyo, Japan).

The Th9 spinous process and vertebral arch were removed, and the dural canal was identified. To establish the F98 and 9L rat SCG models, each cell line, diluted in a 3 μL solution of DMEM with 1.4% agarose (Wako Pure Chemical Industries, Osaka, Japan), was implanted into the intramedullary spinal cord at the Th9/10 level. Using an automated infusion pump, 10^4^ cells or 10^5^ cells were implanted at a rate of 1.5 μL/min into the intramedullary spinal cord using a 26G sharp needle and 25 μL Hamilton syringe (Hamilton Company, Reno, NV)^48,49^. The needle was first inserted at a depth of 3 mm from the dura mater and pulled back to 0.5-mm (2.5 mm from the dura); implantation was initiated at this point. To establish the medium injection models (sham-operated group), a 3 μL solution of DMEM with 1.4% agarose without tumor cells was implanted using the above-described procedure. Finally, the fascia and skin were sutured using 3–0 silk thread. The surgical procedures were performed as described previously^48,49^.

### Basso, Beattie, and Bresnahan (BBB) scale

For functional testing of hind limb muscle strength, we employed the BBB locomotor scale^50,51^. In brief, rats were released into an open-field testing area and observed primarily for lower limb movements for approximately 3 min on a 22-point scale, ranging from 0 (no observed hind limb movements) to 21 (plantar parallel to the trunk, good lower limb clearance, gait coordinated, no trunk sway, and tail always up)^48–51^. All rats exhibited perfect BBB scores prior to tumor implantation. After the procedure, the test was performed daily, starting on the day of implantation (after awakening from anesthesia). Observations were performed by a different researcher and not the practitioner^48^.

### In vivo biodistribution of boron in F98 and 9L rat SCG models

Approximately 6–12 days post-implantation of 10^4^ or 10^5^ F98 rat glioma or 9L rat gliosarcoma cells, when sufficient tumor growth was expected (scored ≤ 5 on the BBB scale), BPA was administered at 250 mg/kg rat body weight (b.w.)^39–42^. At predetermined times (2.5, 6, and 24 h), the rats were euthanized, and relevant tissues (tumor, spinal cord, brain, blood, heart, lung, liver, kidney, spleen, skin, muscle, adrenal gland, and intestine) were harvested. Each organ was weighed and then digested with 1 N nitric acid solution^39–42^. The amount of boron in each organ was measured using ICP-AES. All results (boron concentrations) were defined as μg boron (10B)/g.

### In vivo neutron irradiation of F98 rat SCG models, sham-operated models, and normal rats

The timing of the irradiation experiment was determined based on the survival time and BBB scale of the SCG model. The prerequisite was that all SCG rat models were viable and neurological function recovered from the transplant surgery. Based on this condition, we estimated the time for the microenvironment around the tumor mass to fully develop without significant neurological deficits and determined that 7 days after implantation was appropriate. A total of 37 rats were divided into three neutron irradiation experiments: in vivo neutron irradiation (study 1), neutron irradiation of F98 rat SCG models; in vivo neutron irradiation (study 2), neutron irradiation of medium injection models; in vivo neutron irradiation (study 3), neutron irradiation of normal rats. Study 1 was designed to evaluate the therapeutic efficacy of BNCT in the F98 rat model of SCG. Study 2 was designed to evaluate the safety of the operation and the BBB scale after sham surgery (control group). Study 3 evaluated the safety of the neutron irradiation experiments. In study 1, seven days after implantation of 10^4^ F98 rat glioma cells, 22 rats were divided into the following three groups: group 1, untreated group (untreated); group 2, neutron irradiation control group (neutron-only); group 3, neutron irradiation after 2.5 h of BPA i.v. (BNCT BPA i.v. 2.5 h ). In study 2, seven days after the implantation of DMEM only, 12 medium-injected model rats were divided into the following three groups: group 4, untreated group (sham-untreated); group 5, neutron irradiation control group (sham-neutron-only); group 6, neutron irradiation 2.5 h after BPA intravenous administration (i.v.) (sham-BNCT BPA i.v. 2.5 h ). In study 3, three normal rats were irradiated 2.5 h after i.v. BPA (Normal-BNCT BPA i.v. 2.5 h).

Seven days after tumor cell or medium implantation, a neutron irradiation experiment was conducted at the Institute for Complex Science, Kyoto University. All rats were anesthetized via the intraperitoneal administration of anesthetics, and BPA was administered to the assigned experimental groups. Neutron irradiation was concentrated to the back of the thoracic level; the procure was performed for 20 min at a reactor power of 5 MW and a neutron flux of 9.4 × 10^8^ neutrons/cm^2^/s at the Heavy Water Irradiation Facility at KURNS. All rat bodies exclusively on their back of the thoracic level were shielded with a plate lined with ^6^LiF ceramic tiles to minimize extraneous exposure. All rats were observed until death or euthanasia. We had assumed that the endpoint of experiments in all the in vivo studies was death or BBB scale of 5 or less. In addition, the therapeutic effects were evaluated using Kaplan–Meier survival curves, and the percentage increase in life span (%ILS) was calculated using the following equation: ( median survival time; MST of each BNCT group–MST of untreated group) × 100/(MST of untreated)^39–42,47^.

In the three neutron irradiation experiments, all rats were evaluated using the BBB scale, and survival time was evaluated in the therapeutic neutron irradiation experiment.

### *Estimation of the absorbed dose and photon-equivalent dose *in vivo* neutron irradiation*

The absorbed dose in BNCT was calculated using the following equation: D_B_ + D_N_ + D_H_ + D_γ_^9,41^. D_B_ corresponds to ^10^B(n,α)^7^Li neutron reaction, defined as 7.43 × 10^–14^ (Gy cm^2^/μg ^10^ B/g) × boron concentration (μg ^10^ B/g) × thermal neutron fluence (1/cm^2^). D_N_ corresponds to ^14^N(n,p)^14^C neutron reaction, defined as 6.78 × 10^–14^ (Gy cm^2^/weight %) × nitrogen concentration (weight %) × thermal neutron fluence (1/cm^2^)^9,41^. D_H_ corresponds to ^1^H(n,n)^1^H neutron reaction, defined as the elastic scattering between epithermal or fast neutrons and the hydrogen nucleus. D_γ_ corresponds to ^1^H(n,γ)^2^H neutron reaction, defined as the γ-ray dose resulting from γ-rays emitted when a hydrogen atom captures a thermal neutron and γ-rays from the source itself^41^. Photon-equivalent doses were calculated using the following equation: D_B_ × compound biological effectiveness (CBE) + D_N_ × relative biological effectiveness of nitrogen (RBE_N_) + D_H_ × relative biological effectiveness (RBE_H_) + D_γ_^9,41^. CBE is specific for irradiated tissues and boron compounds^9,39–42^. CBE factors for the normal spinal cord and spinal cord tumors were determined from a previous report: CBE for the normal spinal cord was set as 1.35, and that for spinal cord tumors was set as 3.80, as in the case of BNCT using BPA for intracranial malignant gliomas ^9,36^.

### Statistical analysis

All statistical analyses were performed using the JMP® Pro version 16.1.0. (SAS Institute, Cary, NC, USA). In the i*n vitro* cellular uptake of boron, the boron concentration in each cell was evaluated using Student’s *t*-test. The BBB scale score was calculated as the mean BBB score ± standard deviation for each group. In each group, the BBB score for euthanized animals was considered “0.” The BBB score of each group was evaluated using a multivariate analysis of variance (MANOVA). Kaplan–Meier curves were used to evaluate survival times. Log-rank tests were used to determine significant differences between the groups. For all tests, *P* values < 0.05 were deemed significant.

### Institutional review board statement

This study is reported in accordance with ARRIVE guidelines (https://arriveguidelines.org). This study was conducted in accordance with the guidelines of the Declaration of Helsinki and approved by the Animal Use Review Board and Ethical Committee of Osaka Medical and Pharmaceutical University (No. 21124-A) and the Institute for Integrated Radiation and Nuclear Science, Kyoto University (KURNS; Kumatori, Osaka, Japan) (No. 2022–23).

## Data Availability

The datasets analyzed in this study are available from the corresponding author upon request. The JMP Pro version 16.1.0. software (SAS, Cary, NC, USA) was used for statistical analysis.
